# Physician blame and vulnerability: novel predictors of physician willingness to work with patients who misuse opioids

**DOI:** 10.1186/s13722-021-00242-w

**Published:** 2021-05-25

**Authors:** Berkeley Franz, Lindsay Y. Dhanani, Daniel L. Brook

**Affiliations:** 1 Department of Social Medicine, Heritage College of Osteopathic Medicine, Ohio University, Grosvenor 311, Athens, OH 45701 USA; 2 Department of Psychology, Ohio University, 22 Richland Avenue, Athens, OH 45701 USA; 3 College of Medicine, College of Public Health, The Ohio State University, 250 Cunz Hall, 1841 Neil Ave, Columbus, OH 43210 USA

**Keywords:** Opioids, Medication assisted treatment, Bias, Family medicine, Prescribers

## Abstract

**Background:**

Successfully combating the opioid crisis requires patients who misuse opioids to have access to affirming and effective health care. However, there is a shortage of physicians who are willing to work with these patients. We investigated novel predictors of what might be contributing to physicians’ unwillingness to engage with this patient population to better identify and direct interventions to improve physician attitudes.

**Methods:**

333 physicians who were board certified in the state of Ohio completed a survey about their willingness to work with patients who misuse opioids. The hypothesized relationships between the proposed predictors and willingness to work with this patient population were tested using multivariate regression, supplemented with qualitative analysis of open-text responses to questions about the causes of addiction.

**Results:**

Perceptions of personal invulnerability to opioid misuse and addiction, opioid misuse and addiction controllability, and health care provider blame for the opioid crisis were negatively associated with physician willingness to work with patients who misuse opioids after controlling for known predictors of physician bias toward patients with substance use disorders. Physicians working in family and internal medicine, addiction medicine, and emergency medicine were also more willing to work with this patient population.

**Conclusions:**

Distancing oneself and health care professionals from opioid misuse and placing blame on those who misuse are negatively associated with treatment willingness. Interventions to improve physician willingness to work with patients who misuse opioids can target these beliefs as a way to improve physician attitudes and provide patients with needed health care resources.

**Supplementary Information:**

The online version contains supplementary material available at 10.1186/s13722-021-00242-w.

## Background

The United States remains embroiled in a decades-long syndemic of opioid misuse, opioid use disorder (OUD), and their sequelae [1]. Since 1999, nearly 4,50,000 lives have been lost to unintentional opioid-related overdoses and, though considerable resources have been devoted to combating the crisis, the unintentional opioid overdose rate is still rising in many regions across the country, particularly in the Midwest and Appalachia [2]. Adding to the toll of the opioid crisis, secondary infections caused by shared injection drug equipment have also risen substantially over the last decade, including hepatitis C infections and infective endocarditis [3, 4]. The opioid crisis has also substantially strained the United States health care system both in terms of financial costs and health care resources [5]. Importantly, these downstream consequences of opioid misuse may be preventable with evidence-based therapies such as medications for OUD [6]. Access to medications for opioid use disorder is uneven across the U.S., however, and there is a shortage of physicians who are eligible to prescribe these medications for patients with OUD [7–10].

Opioid misuse refers to “any use outside of prescription parameters, including misunderstanding of instructions, self-medication of sleep, mood, or anxiety symptoms, and compulsive use driven by an opioid use disorder” and we interpret this as including both prescription and illicit opioids [11]. The paucity of available physicians to engage individuals who misuse opioids is critical as this patient population must interface with health care professionals both for OUD treatment and for secondary health conditions stemming from opioid misuse [6]. Indeed, the limited access to health care services due to provider shortages and physicians’ lack of willingness to engage with this patient population [12] has left many counties, especially in rural areas, at an increased risk for an infectious disease outbreak [10, 13]. Although there is evidence of a growing non-traditional addiction medicine provider population among primary care and other specialists [14], these provider populations may not have the same depth of training around the causes and risk factors for opioid misuse and OUD as more traditional addiction treatment providers such as addiction psychiatrists or addiction medicine physicians. Often, health care professionals with the capacity to prescribe medications for OUD do not actually care for patients with OUD [15]. Thus, in order to expand treatment access, evidence is needed to identify what prevents some physicians from being willing to work with patients who misuse opioids see Additional file 1.

Extant studies on physician bias toward patients with substance use disorders (SUDs) and its correlates [16] have demonstrated that negative attitudes toward patients with SUDs are common and that bias represents a significant barrier to health care access [16]. However, there are important limitations in these studies that impair our ability to identify and address barriers to treatment access among patients who misuse opioids. First, studies have predominantly focused on general attitudes toward patients who use and/or misuse drugs and relatively few studies have examined the extent to which physicians are willing to work with patients who misuse opioids in particular. It is important to examine attitudes toward patients who misuse opioids because physicians may have unique experiences and beliefs about this patient population. For example, there is a distinct history between medical professionals and opioid use which may influence attitudes toward this patient population. Second, previous studies have focused on a limited number of correlates that are broadly related to physician bias (e.g., stress, burnout, and contact [16, 17]) and may be failing to capture important determinants of physician willingness to work with patients who misuse opioids. This is again important given that physicians’ experiences with patients who misuse opioids may differ from their experiences with other patient populations, perhaps leading to distinctive correlates of physician bias toward these patients. Thirdly, the recent attempts to eliminate the X-waiver requirement by the US Department of Health and Human Services have the potential to increase access to OUD treatment if successfully passed [18], but the elimination of this barrier may not lead to realized increases in office-based buprenorphine prescribing if physicians remain unwilling to engage with patients who misuse opioids.

The goal of the current paper is therefore to propose and investigate correlates that have not previously been examined to provide a more comprehensive view of the predictors of physician attitudes toward working with patients who misuse opioids. More specifically, we examine perceptions that patients can control their opioid misuse, perceptions that health care providers have contributed to the opioid epidemic, and perceptions that physicians are personally invulnerable to opioid misuse as predictors of willingness to work with patients who misuse opioids. These variables were chosen because of their relevance to the U.S. opioid epidemic wherein prescription opioids and prescribers have played a role in the growing rates of opioid misuse. We posit that physician attitudes toward patients who misuse opioids may be informed by this unique historical context and the corresponding appraisals of health care professionals’ contribution to the opioid crisis. Targeting the reasons behind providers’ willingness to work with patients who misuse opioids may inform future interventions to increase access to care for this underserved patient population.

## Methods

### Study Population

The sample for our study includes 333 board-certified physicians licensed to practice medicine in the state of Ohio, a state with one of the highest unintentional opioid overdose rates [19]. We recruited physicians to participate in a study to better understand how growing opioid misuse has affected physicians working in a state with a high number of unintentional opioid overdose deaths. Potential physician participants were identified with help from the State Board of Medical Licensing which provided a database of contact information for physicians who are licensed to practice in Ohio. Using this information, we emailed all physicians who listed an Ohio address (34,397) and invited them to participate in the study if their medical practice had been “reshaped by the opioid epidemic.” This number represents the total number of physicians who were registered with the state board of medical licensing in Ohio. However, not all of these contacts represent eligible participants. For example, some of the registered physicians may have been retired, changed careers, listed inactive email addresses, or had otherwise out of date records. It is therefore not possible for us to estimate how many of our initial contacts were eligible for the survey or to estimate our initial sampling frame. The first email invitation to participate in the survey was sent in October 2019 and a reminder email was sent three weeks later. The survey remained open through November 2019 until we had reached our a priori goal of 400 participants. We set this a priori goal to ensure that our statistical models were adequately powered and to ensure feasibility for coding a large number of open-ended survey responses. Due to missing data on the quantitative measures, our final sample for the quantitative analysis was 304 participants after employing listwise deletion. We analyzed the open-text responses for all participants who answered this question (n = 333). Participants were not compensated individually but were entered into a raffle to win one of three $200 Amazon gift cards.

Physicians who participated in the study were approximately evenly split between primary care (56%) and specialty practice (44%). The average participant age was 51.43 years old. 60.7% of physician participants identified their sex as male which is comparable to the overall percentage of physicians in Ohio who are male (65%). Participants worked an average of 46.36 h per week and participants had been in their current position for an average of 13.08 years. Approximately 11% of participants had training in addiction medicine, while 44.5% practiced in family or internal medicine (as compared to 24% of physicians working in Ohio). A higher percentage of physicians in our study also worked in emergency medicine (13.6%) as compared to the general physician population in Ohio (5%). The study was approved by the Ohio University internal review board and all respondents provided electronic informed consent prior to participation.

### Data and measures

We used a survey composed of open- and closed-ended questions to assess the extent to which beliefs about the controllability of addiction, perceptions of physicians’ personal invulnerability to addiction, and beliefs about the culpability of health care providers in the opioid crisis shaped willingness to work with patients who misuse opioids. All measures were rated on a response scale of 1 (*strongly disagree*) to 5 (*strongly agree*) and scale composites were computed by averaging responses across the items for each scale. Our dependent variable is physicians’ scores on a five-item scale which was designed to assess willingness to work with patients who misuse alcohol. A longer version of the scale was initially created by Cartwright [20] and we shortened and adapted the scale to measure willingness to work with patients who misuse opioids. Example items from the scale include, “In the future I would accept more patients that are opioid misusers” and “I would enjoy my job more if I could discontinue work with opioid misusers” (reverse coded). Higher scores indicate greater willingness to work with these patients. The adapted scale reliability was α = 0.87. We also examined the factor structure of the adapted scale by conducting an exploratory factor analysis (EFA) using principal axis estimation. Results suggested a single factor with an eigenvalue that exceeded 1.00 and all items had factor loadings that exceeded 0.40 [21].

Our focal independent variables are three measures of beliefs about blame for and vulnerability to opioid misuse and addiction. The first measure is a four-item scale measuring beliefs about whether patients are in control of or are responsible for their addiction. The scale was originally used to measure beliefs about control for people who inject drugs and was created by Brener and von Hippel [22]. We adapted it to focus on patients who misuse opioids and example items include, “Opioid misusers can stop using drugs whenever they want to” and “Opioid misusers have weak characters.” Higher scores indicate higher perceptions of controllability. The EFA for the adapted controllability scale demonstrated a single factor with an eigenvalue greater than 1.00 and all factor loadings were greater than 0.40.

The second measure considered physician beliefs about their own vulnerability to opioids misuse and addiction. Given that no measure was available in the extant literature, we created a six-item scale to measure physician beliefs of invulnerability. The items were: “If I were prescribed opioids, I am confident I could take them and not become addicted;” “Even if facing significant hardship or mental illness, I would not use opioids;” “I would never become addicted to opioids;” “If I experienced urges to continue opioid use after being prescribed an opioid, I would not give in to them;” “If I were prescribed opioids, there is a real risk I would become addicted (reverse scored);” and “It is hard for me to take the perspective of opioid misusers because I know I would never be in their situation.” Higher scores indicate more perceived invulnerability. The physician invulnerability scale developed for this study demonstrated adequate reliability (α = 0.85). Given that this scale was created for the current study, we also conducted an EFA to examine the factor structure. Results identified a single factor with an eigenvalue greater than 1.00. Additionally, one item had a factor loading that fell below 0.40 (i.e., the loading was 0.39). We chose to retain this item because the loading was only slightly below the recommended cutoff.

The final scale was developed to measure beliefs that health care providers contributed to the current opioid crisis. This five-item scale was also created by the authors given the lack of availability of a preexisting scale. The measure included the following items: “Health care providers have been one of the main contributors of the opioid crisis;” “Health care providers get more blame for the opioid crisis than they deserve (reverse coded);” “Even if health care providers did not intend to, they have contributed to the opioid crisis;” “Only a small number of health care providers contributed to the opioid crisis (reverse coded);” and “At most, physicians have played a minor role in the opioid crisis (reverse coded)”. Higher scores indicate that participants perceived health care providers as more responsible for the opioid crisis. This scale also had adequate reliability (α = 0.81). Results from the EFA suggest a single factor with an eigenvalue greater than 1.00 and all factor loadings were higher than 0.40.

#### Control variables

We also controlled for contact with patients with who misuse opioids, stress induced by working with these patients, and burnout given that these variables have previously been found to predict physicians’ willingness to work with patients with SUDs [16, 17, 23]. We assessed physicians’ level of contact with patients using a single item measure which asked physicians to report the percent of their work hours that were dedicated to treating patients who misuse opioids. We measured stress induced by working patients who misuse opioids using a two-item measure that was adapted from von Hippel, Brenner, and von Hippel. Their original scale measured stress induced by working with people who inject drugs [23]. The two adapted items were, “Working with opioid misusers is really a strain for me,” and “Working with opioid misusers directly puts too much stress on me” (α = 0.89). Burnout was measured using the work-related (7 items) and client-related (6 items) subscales of the Copenhagen Burnout Inventory [24] which was designed to measure exhaustion experienced due to one’s job or working with patients (α = 0.91).

We also collected data on physicians’ primary area of practice and recoded them for whether they worked in professional settings in which they may have particular types of contact with patients who misuse opioids. More specifically, we coded their responses as dichotomous variables that reflected whether they worked in addiction treatment, the emergency department, or in family or internal medicine rather than other specialties (e.g., obstetrics, orthopedics). We included the latter designation because family and internal medicine physicians represent the most common opioid prescribers and the highest volume prescribers of any medical specialty [25]. We also controlled for physician demographics including their sex, age, the average number of hours they work per week, and the number of years they have worked in their current positions. Finally, we asked physicians the following open-ended question: “Why do you think your patients misuse opioids?”.

##### Analyses

We examined the relationships between perceived controllability, physician invulnerability, and physician blame and willingness to work with patients who misuse opioids using multiple regression. We first regressed willingness to work with patients who misuse opioids onto the control variables in Step 1 and then added the three focal predictor variables in Step 2. All analyses were conducted in SPSS 27 [26]. We also analyzed the open-ended text responses using Dedoose online software [27]. The first two authors coded all responses independently and met on two occasions to review code applications and ensure intercoder reliability. Coding was initially undertaken to identify major themes, of which blaming physicians who misuse opioids was one of five. Because categories related to physician blame, invulnerability, and perceived controllability emerged within this theme, we then coded all responses to identify whether they related to these topics. Coding agreement was calculated based on how often the authors agreed on the theme into which each response fell. The initial agreement for responses was 86%. This number reflects agreement on how many themes emerged from participant responses. Coders met to discuss any disagreements, including collapsing several themes to reach consensus on the total number of themes.

## Results

### Descriptive statistics

Table 1 displays the means and standard deviations for our quantitative study variables. After excluding participants with missing data, our sample available for the quantitative analyses included 304 physicians. The descriptive statistics for our focal independent variables showed that physicians scored an average of 2.35 (*SD* = 0.85) on perceptions of controllability, an average of 3.52 (*SD* = 0.89) on the invulnerability scale, and an average of 3.19 (*SD* = 0.88) on the physician blame scale. Further, physicians in our sample scored an average of 2.83 (*SD* = 1.05) on the scale measuring willingness to work with patients who misuse opioids.

**Table 1:** Descriptive statistics for sample of physicians surveyed (N = 304)

Variable	N	%	Scale or range	
			Min	Max
Willingness (M ± SD)	2.83 + 1.05		1	5
Blame (M ± SD)	3.19 ± 0.88		1	5
Work Hours (M ± SD)	46.36 ± 16.85		0	100
Gender^a^	187	61	1	2
Tenure (years) (M ± SD)	13.08 + 11.02		0	50
Addiction specialty	35	11	0	1
ER	42	14	0	1
FMIM	137	44	0	1
Contact hours (M ± SD)	18.66 + 20.05		0%	100%
Stress (M ± SD)	3.70 ± 1.18		1	5
Burnout (M ± SD)	2.26 ± 0.79		1	5
Controllability (M ± SD)	2.35 ± 0.85		1	5
Invulnerability (M ± SD)	3.52 ± 0.89		1	5
Age (M ± SD)	51.43 ± 11.84		28	84

*M* mean, *SD* standard deviation, *Min minimal value* Max maximum value, *ER* emergency room, *FMIM* family medicine/internal medicine

^a^number of male participants

We found that perceptions of invulnerability were particularly prevalent, with only 35.1% of our sample scoring at or below the midpoint of the scale. Perceptions of physician blame were more mixed and 43.5% of our sample did not agree that health care providers significantly contributed to the opioid crisis. Finally, our sample reported comparatively low perceptions of controllability and the majority (83.1%) of participants scored at or below the midpoint of the scale.

### Regression results

Standardized results from the regression model assessing the relationship between provider-level characteristics and willingness to work with patients who misuse opioids are shown in Table 2. Results for Step 1 (*R*^*2*^ = 0.403) indicated the only demographic variables that were significantly related to treatment willingness were gender (*b* =  − 0.10, *p* = 0.039) and working in addiction medicine (*b* = 0.18, *p* = 0.001). Further, contact with patients who misuse opioids (*b* = 0.29, *p* < 0.001), stress induced by working with patients who misuse opioids (*b* =  − 0.37, *p* < 0.001), and burnout (*b* =  − 0.16, *p* = 0.002) were significantly related to willingness; contact with patients who misuse opioids was associated with increased willingness and stress and burnout were associated with decreased willingness to work with people who misuse opioids.Results from Step 2 of the model also indicated that perceptions of controllability (*b* =  − 0.15, *p* = 0.002), beliefs that one is invulnerable to opioid misuse and addiction (*b* =  − 0.19, *p* < 0.001), and beliefs about health care provider blame in the opioid crisis (*b* =  − 0.14, *p* = 0.002) were significantly related to willingness to work with patients who misuse opioids. Further, the change in R^2^ for the three variables added in Step 2 was 0.105. These findings suggest that, after controlling for known predictors of physician bias, treatment willingness is lower when physicians believe opioid misuse and addiction are a choice or are within the control of those who misuse or become addicted as well as when physicians have higher beliefs that they would not personally misuse opioids or experience opioid addiction. Conversely, treatment willingness increases when physicians believe health care providers have contributed to the opioid crisis. In addition to addiction medicine physicians, physicians practicing in emergency, family, and internal medicine were more willing to work with patients who misuse opioids in Step 2 as compared to physicians working in other specialties such as OB-GYN, Surgery, and Orthopedics, among others.

**Table 2:** Multivariate predictors of willingness to work with patients with opioid use disorder (n = 304)

Variable	Coef	SE	95% CI		R^2^ (ΔR^2^)
			Lower	Upper	
Step 1					
Age	− 0.083	0.006	− 0.208	0.041	
Gender (1 = male)	− 0.100*	0.103	− 0.194	− 0.005	
Work hours	0.018	0.003	− 0.082	0.118	
Tenure	− 0.050	0.006	− 0.169	0.069	
Addiction	0.179**	0.179	0.075	0.289	
ER	0.070	0.157	− 0.032	0.172	
FMIM	0.083	0.105	− 0.015	0.181	
Contact	0.291***	0.003	0.188	0.399	
Stress	− 0.367***	0.046	− 0.469	− 0.267	
Burnout	− 0.160**	0.068	− 0.261	− 0.059	
					0.403
Step 2					
Age	− 0.034	0.005	− 0.149	0.080	
Gender (1 = male)	− 0.104*	0.094	− 0.191	− 0.017	
Work hours	0.031	0.003	− 0.061	0.123	
Tenure	− 0.032	0.005	− 0.141	0.077	
Addiction	0.150**	0.164	0.055	0.251	
ER	0.115*	0.145	0.021	0.208	
FMIM	0.106*	0.096	0.016	0.196	
Contact	0.281***	0.003	0.186	0.381	
Stress	− 0.297***	0.042	− 0.392	− 0.204	
Burnout	− 0.120*	0.064	− 0.215	− 0.025	
Controllability	− 0.151**	0.059	− 0.245	− 0.056	
Invulnerability	− 0.186***	0.056	− 0.279	− 0.092	
Blame	0.139**	0.053	0.051	0.227	
					0.508 (0.105)

*Coef* standardized regression coefficient, *S* standard error, *95% CI* 95% confidence interval, *ER* emergency room, *FMIM* family medicine/internal medicine

^*^ = p < 0.05; ** = p < 0.01; *** = p < 0.001

### Qualitative themes

Participants were also asked an open-ended question about their beliefs regarding why people become addicted to opioids. After excluding participants who did not respond to this question, 333 responses were coded for themes related to perceived controllability, physician blame, and invulnerability to addiction. Although the majority of physicians underscored the underlying physiological basis for addiction, many physicians also emphasized that addiction was a choice or due to personal weaknesses and that they were not personally vulnerable to opioid misuse and addiction. Further, many participants acknowledged the role that physicians played in prescribing opioid medications for pain relief whereas others externalized this blame by emphasizing the stress placed on physicians to manage pain as the fifth vital sign and the individual characteristics that make certain patients vulnerable to opioid misuse and addiction. Examples of each of our three themes are shown in Table 3 along with the participants’ corresponding scores on the willingness to work with patients who misuse opioids scale.

**Table 3:** Physician perspectives on controllability, invulnerability, and blame (N = 333)

Belief:	Participant excerpt:	Willingness score
Controllability	“They are weak-willed and have arrived at this addiction from their own actions or other platforms…Stop blaming the pharmaceutical companies…can nobody have accountability for their own actions in the United States of America?”	1
	I deal with people with disease who want to stay alive and these people are destroying themselves	1.2
	I care for patients one at a time as they come to the OR, but people who are not ready to help themselves are certainly less rewarding. They can’t really be forced to quit without wanting to be involved in their own care	2
	Like all other decisions in life, drug abuse is a personal choice. No one is forced into a sad life of addiction. The only drug abusers we should be sympathetic to are the ones who are genuinely remorseful for their bad decisions, and are truly seeking help to get clean and sober	3.6
	They are addicted and can't quit without significant help	5
Invulnerability		
	I have some very strong opinions about chronic pain as I live with it on a daily basis. I have had 7 back surgeries over the years and there were periods of time where I was on chronic opiates…I never liked the way I felt on these medications but it was the only way I could not be bedridden. I came to believe that addicts get a different reaction when they take the medication. Yes, I developed tolerance but was able to decrease dosages and get off the medications without issue after surgery	1.4
	I have been prescribed opioids and they make me physically ill so I know I would not get addicted to them and avoid them. Other's have euphoria on use which can lead to repeat use and addiction	3.6
	I am confident that I would never become addicted because I have had opioid following surgical procedures and did not like the way I felt. But I have seen people in my own family and patients who really like the way they feel on opioids and become dependent	3.8
	I am more likely if I fall apart to become an Alcoholic than an opioid user	3.8
Physician blame		
	I believe that patients unwittingly become addicted because they have been given open access to opioids in the past by their physicians without much regard to the addictive potential of the medication	1.4
	But I must point out that I WAS AFRAID OF BEING PUNISHED/FINED in the days of patient satisfaction & Flacc scores. We were THREATENED with monetary punishment if we didn’t treat a patient’s pain adequately	2
	Often, patients are prescribed opiates for legitimate reasons but then habitually use them as a means to blunt other life issues or perceived pain	2.2
	Once addicted, they do not have much choice any more. Many addicted by overprescribing- vulnerable populations were more at risk	4

## Discussion

The goal of the current paper was to expand our understanding of barriers to health care access for patients who misuse opioids, especially physician willingness to work with this patient population. We proposed three novel factors that may shape physician willingness to work with patients who misuse opioids: perceptions that opioids misuse and addiction are controllable, perceptions of one’s personal invulnerability to opioid misuse and addiction, and perceptions of prescribers’ culpability in the opioid crisis. These beliefs were all significantly related to willingness to work with patients who misuse opioids. Specifically, believing opioid misuse and addiction to be a product of personal choices or failings decreased treatment willingness whereas believing opioid misuse and addiction to be a product of more systemic actions from the health care system increased treatment willingness. These findings align with previous studies that have linked perceptions of controllability to prejudicial attitudes toward a number of stigmatized groups, including people who inject drugs [17, 28]. Extending previous studies on physician bias toward patients who misuse opioids and those with OUD, however, these findings demonstrate how perceptions of the health care system’s role in perpetuating addiction may reflect the unique historical context and relationship between prescription medications and the opioid crisis in the United States. We posit that physicians who recognize the structural or upstream causes of the opioid crisis are more willing to work with patients who misuse opioids because they are more aware of the broader context that may have contributed to patients’ misuse of opioids. This finding is important because it underscores how personal beliefs among physicians, which may serve a protective function in preserving physicians’ positive job-related self-esteem, can create barriers to patients accessing important health care services.

Our results further demonstrated that many physicians feel invulnerable to opioid misuse and addiction and that these beliefs are associated with decreased willingness to work with patients who misuse opioids. Beliefs of invulnerability, importantly, were relatively common among physicians as compared to the two other measured beliefs. The finding that perceptions of invulnerability are associated with willingness to work with specific patients has not been identified in previous work but comports with research which has demonstrated the importance of empathy in bias reduction [29]. Feeling as though one could not find themselves in the position of patients who misuse opioids may limit perspective taking and physicians’ ability to empathize with these patients. Of even more significant concern, feeling invulnerable to opioid misuse and addiction may reinforce beliefs that addiction is a choice and may limit willingness to participate in evidence-based interventions to prevent and treat opioid misuse. Future research should specifically assess the extent to which feelings of invulnerability relate to prescribing patterns and willingness to administer medications for opioid use disorder such as buprenorphine.

The findings from the current study can also inform future interventions aimed at improving physicians’ attitudes toward patients who misuse opioids by identifying specific and previously unexamined beliefs that underlie or contribute to physician bias. Drawing on our findings, future intervention efforts should seek to increase physicians’ ability to take the perspective of patients who misuse opioids, reduce perceptions that patients are personally responsible for their opioid misuse and addiction, and increase awareness of the structural factors that contribute to the opioid crisis. Importantly, these areas are not the focus of current interventions to reduce bias among physicians which primarily provide education and increase contact with patients with SUDs. Our findings suggest that interventions that successfully change perspectives on the underlying causes of opioid misuse in particular may improve physician willingness to work with patients who misuse opioids and resolve critical barriers to combating the ongoing opioid crisis.

### Limitations

Our study has limitations that are important to acknowledge. First, we cannot draw causal conclusions regarding the relationships tested in this study due to our data being collected in a cross-sectional survey using a convenience sample. Second, although anonymous online surveys are advantageous because they have a lower risk of social desirability bias in response to sensitive questions, this modality also comes with lower response rates. As a result, our sample may not be representative of the full sampling frame from which we drew. We incentivized participants to increase the response rate but, given the high median salary of the survey population, our incentive may not have been desirable enough for many. We also received a few email “bounce-backs” suggesting that the database included some incorrect or outdated email addresses. Because of the qualitative coding of open-ended responses, it was necessary to limit the overall number of participants in the study which further limited the sample size. Thirdly, our survey addressed working with patients who misuse opioids. Opioid misuse can include any use of an opioid in a non-prescribed manner [11]. Therefore, working with patients who misuse opioids could include a range of interventions from preventive measures to address opioid misuse to treatment for OUD. But, because opioid misuse is not a medical diagnosis (as opposed to OUD), leaving opioid misuse undefined may have resulted in information bias whereby respondents did not have a clear understanding of the patient population they were asked about. Not defining opioid misuse and what categorizes working with patients who misuse opioids may limit the generalizability of our findings to a willingness to work with a specific, definable patient population. A final limitation is that not all physician participants answered all survey questions which required excluding these participants from the present analyses. Despite these limitations, we note that our sampling strategy offers important advantages as compared to previous studies on physician bias which have utilized small, homogenous samples.

## Conclusions

Examining the extent to which physicians are willing to work with patients who misuse opioids is essential given the critical need for this patient population to engage with both treatment and general medical services. Our results suggest that distancing oneself and health care professionals from people who misuse opioids and believing opioid misuse is a personal choice is negatively associated with treatment willingness. Intervention efforts should attempt to mitigate these attitudes by recognizing the structural characteristics that contributed to the opioid crisis, increasing perspective taking, and challenging perceptions that only certain individuals are vulnerable to opioid misuse and addiction.

## Supplementary Information

**Additional file1:** Measure of willingness to work with patients who misuse opioids.

## Data Availability

The datasets used and/or analysed during the current study are available from the corresponding author on reasonable request.
