# Supplementary material for: Physician blame and vulnerability: novel predictors of physician willingness to work with patients who misuse opioids
Source: Addict Sci Clin Pract. 2021 May 25;16:33. doi: 10.1186/s13722-021-00242-w (PMC8147073; doi:10.1186/s13722-021-00242-w)
Supplement: Supplementary file 1 — Additional file1: Measure of willingness to work with patients who misuse opioids. [file 13722_2021_242_MOESM1_ESM.docx]

**Appendix**

**Measure of Willingness to Work with Patients who Misuse Opioids**

1. In the future, I would accept more patients that are opioid misusers.
2. I am interested in expanding my expertise in how to work with patients who misuse opioids.
3. I would find it fulfilling to have the opportunity to work with patients who misuse opioids.
4. I want to work with patients who misuse opioids in the future.
5. I would enjoy my job more if I could discontinue working with patients who misuse opioids (reverse scored).
